# Integrating an infectious disease programme into the primary health care service: a retrospective analysis of Chagas disease community-based surveillance in Honduras

**DOI:** 10.1186/s12913-015-0785-4

**Published:** 2015-03-24

**Authors:** Ken Hashimoto, Concepción Zúniga, Jiro Nakamura, Kyo Hanada

**Affiliations:** Takemi Program in International Health, Harvard T.H. Chan School of Public Health, Boston, MA USA; Chagas Disease Control Project 2008–2011, Japan International Cooperation Agency, Tegucigalpa, Honduras; Ministry of Health, Tegucigalpa, Honduras; Project Management Direction, External Cooperation Department, Estrella Inc., Tokyo, Japan; Independent consultant, Chiba, Japan; Former Senior Advisor, Human Development Department, Japan International Cooperation Agency, Tokyo, Japan

## Abstract

**Background:**

Integration of disease-specific programmes into the primary health care (PHC) service has been attempted mostly in clinically oriented disease control such as HIV/AIDS and tuberculosis but rarely in vector control. Chagas disease is controlled principally by interventions against the triatomine vector. In Honduras, after successful reduction of household infestation by vertical approach, the Ministry of Health implemented community-based vector surveillance at the PHC services (health centres) to prevent the resurgence of infection. This paper retrospectively analyses the effects and process of integrating a Chagas disease vector surveillance system into health centres.

**Methods:**

We evaluated the effects of integration at six pilot sites in western Honduras during 2008–2011 on; surveillance performance; knowledge, attitude and practice in schoolchildren; reports of triatomine bug infestation and institutional response; and seroprevalence among children under 15 years of age. The process of integration of the surveillance system was analysed using the PRECEDE-PROCEED model for health programme planning. The model was employed to systematically determine influential and interactive factors which facilitated the integration process at different levels of the Ministry of Health and the community.

**Results:**

Overall surveillance performance improved from 46 to 84 on a 100 point-scale. Schoolchildren’s attitude (risk awareness) score significantly increased from 77 to 83 points. Seroprevalence declined from 3.4% to 0.4%. Health centres responded to the community bug reports by insecticide spraying. As key factors, the health centres had potential management capacity and influence over the inhabitants’ behaviours and living environment directly and through community health volunteers. The National Chagas Programme played an essential role in facilitating changes with adequate distribution of responsibilities, participatory modelling, training and, evaluation and advocacy.

**Conclusions:**

We found that Chagas disease vector surveillance can be integrated into the PHC service. Health centres demonstrated capacity to manage vector surveillance and improve performance, children’s awareness, vector report-response and seroprevalence, once tasks were simplified to be performed by trained non-specialists and distributed among the stakeholders. Health systems integration requires health workers to perform beyond their usual responsibilities and acquire management skills. Integration of vector control is feasible and can contribute to strengthening the preventive capacity of the PHC service.

## Background

### Integration of disease-specific programmes

The effectiveness of integration of disease-specific programmes into primary health care (PHC) services has been challenged for decades [1]. Vertical programmes implement disease control operations through direct command lines, but create additional parallel lines requiring greater financial and human resources [2]. Research findings on the effects of integration have been inconclusive mostly because of the uniqueness of each setting and lack of clear evidence [3-6]. However, it has been observed that disease-specific programmes are more likely to be integrated into PHC services when tasks are clinical [7]. Indeed, integration efforts have focused mostly on clinically oriented disease control programmes such as for HIV/AIDS and tuberculosis and rarely on vector control [3-5,8-11].

### Structural changes of vector control programmes

Vector control programmes are organised in a vertical structure in most countries. Among the earliest, National Malaria Eradication Programmes were launched in malaria endemic countries on the global scale in the 1950s and 1960s with centralised administration to insure implementation of WHO guidelines in a standardised manner [12]. In the 1970s and 1980s, various countries established National Vector Control Programmes, incorporating other vector-borne diseases, such as dengue and onchocerciasis. In Latin America, subsequent health system reform since the 1990s decentralised financial and operational responsibilities of vector control programmes to the local health system [13]. In Honduras, the Vector Control Programme was merged with Zoonosis, Food Security and Basic Sanitation Programmes at the local level to establish the Environmental Health Programme, reducing the total number of operational personnel [14].

### Chagas disease

Chagas disease is a vector-borne disease, caused by the protozoan *Trypanosoma cruzi*, that can lead to potentially fatal chronic cardiomyopathy or disabling megaesophagus and megacolon [15]. In Latin America, approximately 8 million people are infected and 109 million are at risk for infection [16]. Vulnerable populations mostly live in rural and impoverished areas, since more than 80% of the cases are transmitted by vector bugs that preferentially infest mud walls and thatched roofs [17].

### Chagas disease vector control in Honduras

The prevalence of Chagas disease in Honduras decreased from 300,000 in the early 1990s to 220,000 in 2005, following the political advocacy of Central American Chagas Disease Control Initiative (IPCA) launched in 1997 and the country’s successful vector control interventions [16,18,19]. Extensive insecticide spraying campaigns were directed by the National Chagas Programme and were managed by the Environmental Health Programme in the departments. The Environmental Health Programme coordinated with other units in the Departmental Health Office and the local PHC services (health centres), but implemented directly the spraying campaigns with the technical direction of the National Chagas Programme. Houses were sprayed by trained community members under the supervision of municipal and/or departmental Environmental Health technicians. Of the two main vectors, *Rhodnius prolixus* was eliminated in most areas, and *Triatoma dimidiata* was reduced to a controllable level in western Honduras [14,20]. To maintain the risk of infection at a minimum level throughout broad endemic areas, the Honduran Ministry of Health implemented a vector surveillance system consisting of vector bug reporting by the community and institutional response to the reports at local health centres.

This paper retrospectively analyses the effectiveness and the process of establishing a Chagas disease vector surveillance system at health centres in Honduras during 2008–2011 to determine the feasibility of integration of vector control into PHC services.

## Methods

### Pilot sites

Six pilot sites were selected in four endemic departments, Ocotepeque, Copán, Lempira and Intibucá, in western Honduras in 2008, where the Honduran Ministry of Health had extensively sprayed houses and reduced vector infestation during 2004–2007 [19]. The geographic area of a pilot site was defined as the jurisdiction of a health centre. The six pilot sites were selected for their history of high infection risks (presence of *R. prolixus* or household infestation rate of *T. dimidiata* greater than 20%) of Chagas disease but varied in geography, demography, history of vector infestation, health service staff and community personnel (Table 1).

**Table 1:** **Geographic, demographic, entomological and human resource data of the six pilot sites in western Honduras**

**Department**		**Ocotepeque**	**Copán**		**Lempira**	**Intibucá**	
**Health centre**		**San José de la Reunión**	**Rincón del Buey**	**Corquín**	**Santa Cruz**	**Dolores**	**San Marcos de Sierra**
Jurisdiction area (km^2^)		14.0	33.5	138.6	150.0	82.6	142.8
Number of population	Total	471	4,208	11,537	5,862	4,805	5,624
	Principal ethnicity	Indigena (Chortí)	Indigena (Chortí)	Ladino	Indigena (Lenca)	Indigena (Lenca)	Indigena (Lenca)
Number of villages	Total	6	13	45	40	19	34
	With a history of *R. prolixus*	6	2	0	5	8	3
	With a history of *T. dimidiata*	6	11	35	12	19	34
Number of houses	Total	112	676	3,337	1,179	925	995
Number of health centre staff	Physicians	0	1	1	1	2	1
	Professional nurses	0	0	2	1	0	0
	Assistant nurses	1	2	14	2	2	3
	Environmental Health technician	1*	1*	1	1*	1	1
Number of village personnel	Health volunteers	32	30	15	68	48	41
	Trained sprayers	6	12	20	20	40	25

*Part-time.

### Implementation of the vector surveillance system

The Honduran Ministry of Health designed, implemented and evaluated the vector surveillance system in the six pilot sites with the assistance of the Japan International Cooperation Agency (JICA) as part of a bilateral project from 2008 to 2011 [20].

First, the National Chagas Programme, with collaboration of the National Chagas Laboratory, Medical Entomology Unit and aid agencies, drafted a provisional surveillance guideline outlining indispensable tasks at the national, departmental and local levels. To promote and respond efficiently to vector bug reports from inhabitants, the managerial focal point of the surveillance system was placed at the local health centres with a full-time or part-time Environmental Health technician (Table 1).

Second, training was conducted by the National Chagas Programme (technicians) for the Departmental Health Offices (Epidemiologists and Environmental Health Coordinators), who in turn trained health centre personnel (physicians, nurses and Environmental Health technicians), who trained community health volunteers who oriented the inhabitants. The head of each health centre (physician or nurse) was responsible for integration of vector surveillance into the routine work systems. Responsibilities of health centre staff included; promoting vector search; registering bugs reported by the inhabitants; organising response with the community health volunteers; and supplying educational materials, spraying equipment and insecticide to the trained community sprayers. The materials and equipment were provided by the National Chagas Programme through the Departmental Health Office.

Third, surveillance activities were monitored by quarterly visits of the Environmental Health Programme officers in each department and quarterly workshops organised by the National Chagas Programme with assistance of JICA advisors. In the workshops, staff from the six pilot health centres and corresponding Departmental Health Offices presented progress data [20].

### Indicators and data collection

The bilateral project team, consisting of the National Chagas Programme officials and JICA advisors, evaluated progress and effect of surveillance activities in terms of; surveillance system performance; knowledge, attitude and practice (KAP); community bug reports and institutional response to the reports; and seroprevalence.

The surveillance system performance index is a checklist of 48 items composed of indispensable tasks of the National Programmes (11 items), Departmental Health Offices (12 items), health centres (14 items) and communities (11 items) [20]: it was designed by the bilateral project team and based on the provisional surveillance guidelines to analyse the task completion and pitfalls. Examples of performance indicators were quarterly monitoring of the surveillance system by the National Chagas Programme; quarterly consolidation of data by the Departmental Health Office on community bug report and institutional response; organisation of health promotion by health centre staff; and promotion by community health volunteers of bug searches by the inhabitants. Scores were evenly weighted and totalled to 100 points. The assignees evaluated biannually their own performance with the supervisors. Although the content of the checklist was modified slightly at revisions, the results were comparable throughout five evaluations during 2009–2011.

The KAP test was a seven-item questionnaire developed by the bilateral project team to survey knowledge, attitude and practice related to Chagas disease and its prevention among schoolchildren. ‘Knowledge’ items included identification of the vectors and affected organs. ‘Attitude’ focused on whether the children found the vectors harmful. ‘Practice’ questions asked whether the children searched for bugs in their houses in the last six months, and what they did if they found them. Pilot health centre personnel were instructed to test children in the fourth to sixth grade with the KAP test in at least three schools of the jurisdiction before and after a year of the health promotion for Chagas disease surveillance.

The community bug reports and institutional response to the reports were registered at each pilot health centre by an Environmental Health technician, nurse or physician. The registry recorded the name of family chief of infested houses, dates of reception of reports, village names, vector bug species, dates of response, and the name of responder for each report. These data were consolidated and presented at the quarterly workshops.

The seroprevalence survey targeted all children under 15 years-old in each pilot site. The baseline was obtained as part of the previous bilateral project between Honduran Ministry of Health and JICA during 2004–2007 in all sites except Santa Cruz, Lempira [19]. For both baseline and evaluation surveys, the same methods were utilised for sampling and laboratory diagnosis. For collection of blood samples, community health volunteers in each village assembled children under 15 years of age in a public facility (usually primary school), and health centre staff with assistance of Departmental Health personnel collected finger prick blood samples on filter paper (Whatman Grade 1). The samples were dried and sent to the National Chagas Laboratory, where the dried samples were eluted and examined using ELISA recombinant test kits (Weiner Lab Chagatest).

### Data analysis

**Figure 1:**
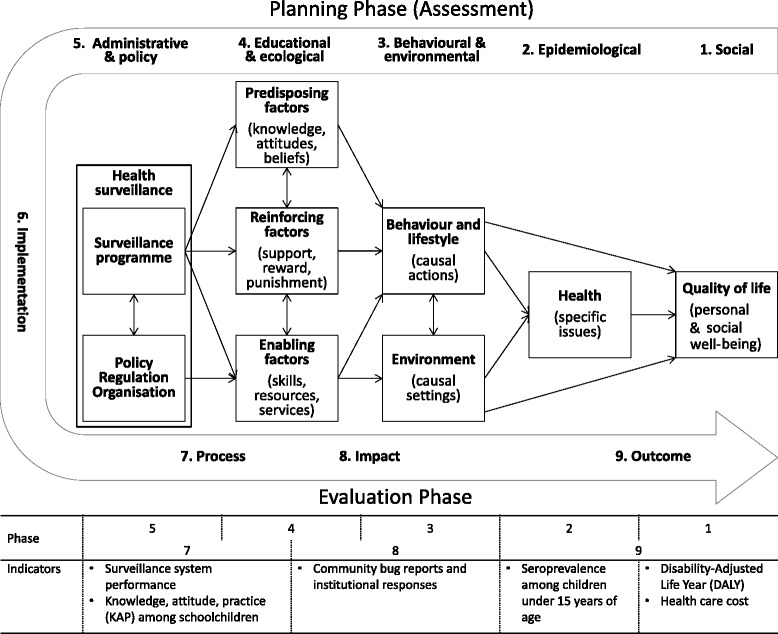
**The PRECEDE-PROCEED model for health programme planning adapted to analysis of Chagas disease surveillance system in PHC services.**

We analysed the process and effects of implementation of the vector surveillance system in the health centres using the PRECEDE-PROCEED (predisposing, reinforcing, and enabling constructs in educational/ecological diagnosis and evaluation – policy, regulatory, and organisational constructs in educational and environmental development) model for health programme planning [21]. PRECEDE is a framework designed to assess health-related behaviours and environment from epidemiological, social, behavioural, educational, administrative and political perspectives. The subsequent PROCEED stage allows planning, implementation and evaluation of public health interventions based on the assessment (Figure 1). We considered the establishment of Chagas disease surveillance systems at health centres as health programme planning, and we hypothesized that the model would facilitate a holistic and systematic analysis for determining key factors at different administrative levels of the Ministry of Health and the community.

To select suitable, influential and interactive factors for the PRECEDE-PROCEED model, the authors reviewed pertinent situations, activities, resources, systems, strategies and policies in published documents [14,19,20]. Because of unavailability of local data on mortality, morbidity and health expenditure related to Chagas disease, the social burden of Chagas disease in terms of Disability-Adjusted Life Year (DALY) and health care costs were estimated referring to a published work [22].

All data were used with the permission of the Ministry of Health of Honduras.

## Results

### Effects of surveillance implementation at health centres

#### Surveillance system performance

**Figure 2:**
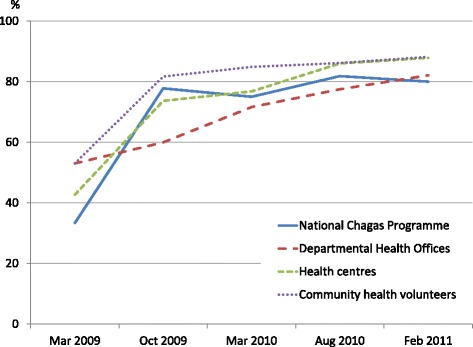
**The mean performance index of the six pilot sites for the Chagas disease vector surveillance system by the National Chagas Programme, Departmental Health Offices, health centres and community health volunteers from 2009 to 2011.**

The surveillance system performance improved throughout national, departmental, health centre and community levels in all six pilot sites. The average score increased from 46 in March 2009, 73 in October 2009, 77 in March 2010, and 83 in August 2010 to 84 in February 2011 (Figure 2). The health centres, which served as the managerial focal points of surveillance system, recorded 43, 74, 77, 86 and 88 in the respective evaluations. Common deficiencies noted were risk map updating, monthly data reporting to the upper administrative offices, and timely response to the bug reports.

#### Knowledge, attitude and practice

Of the six pilot sites, three (Rincón del Buey, Santa Cruz and Dolores) conducted the KAP survey with fourth to sixth grade children (Santa Cruz also included secondary schoolchildren in pre- and post-surveys). The survey results showed significant improvement in attitude - risk awareness - in the three sites, elevating the mean score from 77.4 to 82.7 between July 2009 and October 2010 (Table 2). Knowledge score also increased significantly among the schoolchildren in Dolores, where the health centre organised a health promotion campaign on Chagas disease prevention in 2009.

**Table 2:** **Results of KAP tests on Chagas disease surveillance among schoolchildren in the three pilot sites**

**Department**	**Pilot site**	**Number of participants**		**Disease symptom knowledge** ^**1**^		**Vector bug risk awareness** ^**2**^		**Bug search behaviour** ^**3**^	
		**Jul 2009**	**Oct 2010**	**Jul 2009**	**Oct 2010**	**Jul 2009**	**Oct 2010**	**Jul 2009**	**Oct 2010**
Copán	Rincón del Buey	157	149	80.5	78.5	77.9	83.9*	37.8	44.3
Lempira	Santa Cruz	70	148	75.7	71.0	70.0	75.8**	40.0	51.4
Intibucá	Dolores	314	359	69.1	78.3*	77.4	82.7*	63.2	62.1

^1^ *x* ^2^ test analysed whether the typical disease symptom was identified before and after the health promotion.

^2^ANOVA test compared the scores of vector identification test before and after the health promotion.

^3^ *x* ^2^ test examined whether the bug search was carried out before and after the health promotion.

*p <0.01, **p < 0.05.

#### Community bug report and institutional response

Inhabitants of the six pilot sites sent vector bugs caught in houses to the local health centre (Table 3). *T. dimidiata* was found in all sites, but *R. prolixus* was reported only once, in San Marcos de Sierra. As response to the bug reports, the health centres organised insecticide spraying and educational advice, either within two to three months or after several months of having accumulated a certain number of reports, based on agreement with the community health volunteers and the departmental supervisors. In some cases, neighbouring houses at risk of infestation were also sprayed to prevent possible spread of vectors.

**Table 3:** **Entomological indicators of the six pilot sites from 2008 to 2010**

**Department**	**Pilot site**	**Number of houses reported with** ***R. prolixus***			**Number of houses reported with** ***T. dimidiata***			**Number of houses sprayed**		
		**2008**	**2009**	**2010**	**2008**	**2009**	**2010**	**2008**	**2009**	**2010**
Ocotepeque	San José de la Reunión	0	0	0	14	12	3	8	32	2
Copán	Rincón del Buey	0	0	0	0	50	15	0	0	58
	Corquín	0	0	0	17	35	3	0	0	271
Lempira	Santa Cruz	0	0	0	8	42	10	50	130	15
Intibucá	Dolores	0	0	0	79	122	13	11	376	0
	San Marcos de Sierra	0	1	0	53	74	87	0	0	699

#### Seroprevalence

The mean seroprevalence among the children under 15 years of age at the pilot sites significantly decreased from 3.4% in 2004–2007 to 0.4% in 2010 (Table 4). Corquín had recorded low seroprevalence at the baseline survey, due to absence of *R. prolixus,* the more efficient vector, and did not conduct the evaluation survey.

**Table 4:** **Serological indicators of the six pilot sites for pre- and post- intervention**

**Pilot site**	**Seroprevalence in children < 15 years-old % (No. positive/No. sample)**	
	**2004-2007**	**2010**
San José de la Reunión	4.2 (15/356)	0* (0/174)
Rincón del Buey	10.5 (54/512)	0.6* (2/313)
Corquín	0.2 (3/1,351)	NA
Santa Cruz	NA	0.3 (6/2,345)
Dolores	3.0 (58/1,943)	0.2* (1/481)
San Marcos de Sierra	4.4 (57/1,293)	2.0** (6/298)

Using *x* ^2^ test: *p < 0.01, **p < 0.05.

NA Not Available.

#### Disability Adjusted Life Years (DALYs) and health care cost

Decline in the total number of Chagas disease cases from 95 to eight among the children under 15 years of age in the six pilot sites (Table 4) is also considered to have reduced DALYs and health care costs by 92%. Assuming that a patient with chronic Chagas disease annually adds 0.51 DALYs and US$383 of health care cost on average [22], a total of 88 DALYs and US$65,876 were saved respectively on a yearly basis.

Similarly if the seroprevalence decreased at the same rate for all populations in the six pilot sites from 2008 to 2010, the Honduran Ministry of Health would have saved US$1,125,241 during the three year period with US$12,121 direct investment, consisting of US$10,087 for the insecticides (US$6.11 per house) and US$2,034 for the quarterly monitoring by the departmental supervisors (US$24.14 of labour and US$4.11 of fuel per visit). This indicates that a dollar investment yielded US$93 saving in the health care cost.

### Factors associated with surveillance implementation at health centres

#### Influential and interactive factors

**Figure 3:**
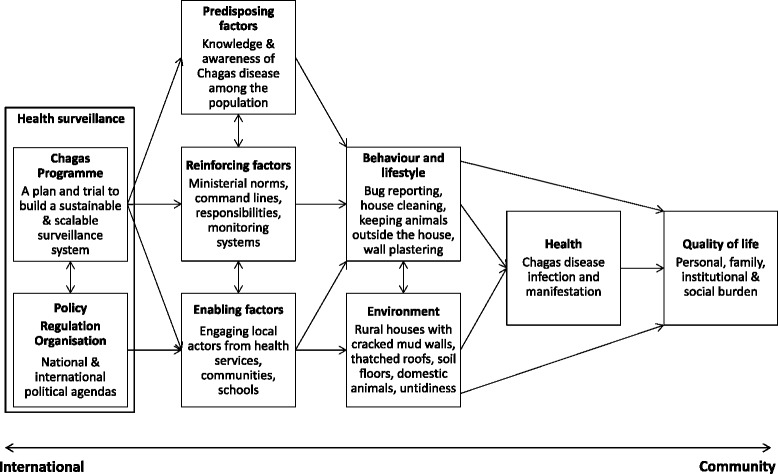
**Key factors interactively associated with the community-based vector surveillance system for Chagas disease within the PRECEDE-PROCEED framework.**

Using the PRECEDE-PROCEED model, we identified the most influential and interactive factors from the community to international level in establishing the Chagas disease vector surveillance system at health centres (Figure 3).

The bottom-up PRECEDE assessment determined essential factors and resulting situations at or near the community level (as seen in the indicators in Figure 1). The health centres (Enabling factors in Figure 3) influenced the behaviour, lifestyle and living environment of inhabitants directly and via community health volunteers found in all villages. The communication between health centre staff and community health volunteers was established through monthly meetings, but Chagas disease was not included in their routine surveillance topics. Further, although the health centres were organisationally positioned under the Departmental Health Office, the command line was not available for vertical interventions because of the decentralised and independent administration of health centres, subdivided PHC services and established health services network within or between municipalities.

At the PROCEED stage, the National Chagas Programme (indicated as “Chagas Programme” in Figure 3) programmed and directed the surveillance implementation by enabling and reinforcing the local health service. In accordance with the assessment outcomes, the National Chagas Programme assigned monitoring responsibilities to the Departmental Health Offices and operational management to the health centres, and prepared corresponding tasks, training and evaluation schemes to develop capacities including knowledge, skills and systems (Table 5). At the same time, to design a scalable surveillance model for endemic areas with distinct vector distribution and institutional response capacity, the National Chagas Programme requested feedback from all pilot sites in particular to improve the practicality of surveillance guidelines. The whole process of design, implementation and evaluation was reinforced by national and international political, administrative and technical supports (Table 5).

**Table 5:** **Key factors and their potential contribution to establishment of the Chagas disease vector surveillance at the PHC service**

**Administrative level**	**Key factors**	**Possible contribution (Directly influenced factors in Figure ** 3 **)**
Central American Chagas Disease Control Initiative (IPCA)	• IPCA coordinated by PAHO/WHO conditioned implementation of community-based vector surveillance as criteria for certifying the interruption of Chagas disease vector-borne transmission [18,25].	Political advocacy (Chagas Programme)
National Chagas Programme	• The National Strategic Plan 2008–2015, which aimed to design and scale up a sustainable surveillance system throughout the endemic areas [26].	Political advocacy (Chagas Programme)
	• A bilateral project 2008-2011with JICA, which aimed to establish a sustainable and scalable surveillance system and provided political, managerial and technical supports [20].	Political advocacy, technical and managerial support (Chagas Programme)
	• Leadership to involve different National Programmes, donors, Departmental Health Offices and to mobilise resources.	Administration (Chagas Programme)
	• Projection of visible surveillance design by provisional guidelines with indispensable tasks for the national, departmental and local levels.	Technical and managerial alignment (Chagas Programme)
	• Cascade training, followed by trial and error approach to continue improving the surveillance model.	Development of skills and models (Enabling factors)
	• Provision of technical support, monitoring and evaluation.	Improvement of systems and performance (Reinforcing factors)
Departmental Health Office	• Cascade training of health centre staff and monitoring of the surveillance system performance.	Improvement of skills, systems and performance (Enabling & reinforcing factors)
	• Assignation of the head of health centre as responsible for the surveillance system, to manage and integrate into the routine systems.	
Health centre	• Training of community health volunteers, stakeholder analysis and task distribution to implement the community-based surveillance [14].	Improvement of skills, systems and performance (Predisposing & enabling factors)
	• Management of the surveillance data, materials, staff and community health volunteers to provide timely response to the bug reports.	
Community health volunteer	• Orientation of the inhabitants, community leaders and schoolteachers on bug surveillance and disease prevention.	Improvement of knowledge and community empowerment (Predisposing & enabling factors, behaviour, lifestyle, environment)
	• Exchange of information on progress with health centre staff during the monthly meetings.	

## Discussion

We found that Chagas disease vector surveillance can be integrated into the PHC services after the vertical interventions. The results showed clear improvement in surveillance system performance, knowledge and attitude among schoolchildren, and seroprevalence in children under 15 years of age. We also identified key factors which facilitated the implementation and management of the surveillance system at the national, departmental, health centre and community levels.

While previous integration efforts focused on clinically oriented disease control programmes, our research investigated the feasibility of integrating a vector control programme into PHC services. Integration was achieved by designing a practical surveillance model and adequately involving multilevel stakeholders to play their individual roles (e.g. promoting bug searches, sending bugs to health centres, monitoring surveillance tasks, and providing materials) in such a way to continue improving the performance (Figure 2).

Research has shown that health care tasks can be simplified, standardised and shifted to less specialised personnel, for example from physicians to nurses and health professionals to lay providers, in settings with limited human resources [6,12]. Our findings suggest that seemingly highly technical vector surveillance tasks can also be simplified with little increase in workload to be incorporated into the PHC services.

Vector surveillance may not be considered the direct responsibility of the head (physician or nurse) of PHC services, particularly in countries with a well-established vector control programme. However in case of Honduras with limited operational personnel, by assigning the head of health centres as the manager responsible for Chagas disease vector surveillance, the surveillance tasks were distributed and integrated into the routine work according to each setting, as observed in different response patterns for community bug reports. Health systems integration requires health workers to perform beyond their area of expertise and acquire adequate management skills [12,23]. The Honduran experience suggests that the head of PHC services when trained, given operational responsibility and monitored can develop capacities to manage different disease control activities including vector surveillance.

Several key factors were identified using the PRECEDE-PROCEDE framework. The PHC services functioned rather independently from the Departmental Health Offices in comparison to vertical programmes, and influenced the population directly and via community health volunteers. The National Chagas Programme suitably implemented the surveillance system by assigning operational management to the health centres and monitoring responsibility to the Departmental Health Offices. Also, participatory revision of the surveillance guidelines with constant feedback by stakeholders from different levels and from distinct pilot sites - trial and error -, facilitated construction of a practical and scalable model as well as development of skills, knowledge and ownership of the participants. Indispensably the whole integration process was supported by national and international political advocacy.

Our retrospective analysis on the Honduran field experience provided realistic results and lessons. In fact, the vector surveillance model established at the six pilot sites was scaled up to other endemic areas in the country [20]. However, although the results of serological surveys showed significant reduction of prevalence of infection, the post-intervention data may represent the mixed effects of massive insecticide spraying campaigns during 2004–2007 and surveillance activities of 2008–2010. Targeting children under 15 years of age for both surveys may have excluded some of the most highly exposed individuals in the post-intervention evaluation. Also because of lack of information on morbidity, mortality and expenditure of the local health services related to Chagas disease, our research was unable to provide a robust analysis of social burden and cost-effectiveness.

Future research is suggested to address the long-term effects of vector surveillance integrated into PHC services in terms of performance, cost-effectiveness, quality and coverage, as well as outcomes including seroprevalence and household vector infestation rate. Comparison of these dimensions between integrated and vertical surveillance approaches may allow further understanding of the effectiveness of integration.

In improving public health services, one of the major challenges is shift from cure to prevention. Considering that vector surveillance is a preventive measure manageable at PHC services, integration efforts can strengthen local health systems and thereby reduce health care costs in the long run. We believe that the Honduran experience would serve as a reference for those in positions to operationalise the integration of specific-disease control programmes. Developing the capacity of PHC services however should not compromise the number of frontline health workers already in serious shortage [24], rather should elevate the overall performance by optimizing available resources and mechanisms.

## Conclusions

We found that integration of Chagas disease vector surveillance into the PHC service was feasible. The health centres demonstrated capacity to manage the community-based surveillance system in terms of performance, knowledge and awareness of schoolchildren, vector report-response, and seroprevalence, once the tasks were simplified to be performed by trained non-specialists, and collaborated actively by multilevel stakeholders. While future studies should evaluate the long-term effects of integration, this research on the Honduran experience outlines a potential strategy for strengthening vector surveillance and therefore disease prevention capacity of local health systems.

## Abbreviations

JICA: Japan International Cooperation Agency
KAP: Knowledge, attitude and practice
PRECEDE-PROCEED: Predisposing, reinforcing, and enabling constructs in educational/eco logical diagnosis and evaluation – policy, regulatory, and organisational constructs in educational and environmental development
